# Rapid remodeling observed at mid-term in-vivo study of a smart reinforced acellular vascular graft implanted on a rat model

**DOI:** 10.1186/s13036-022-00313-9

**Published:** 2023-01-03

**Authors:** Francis O. Obiweluozor, Mukhammad Kayumov, Yujin Kwak, Hwa-Jin Cho, Chan-Hee Park, Jun-kyu Park, Yun-Jin Jeong, Dong-Weon Lee, Do-Wan Kim, In-Seok Jeong

**Affiliations:** 1 Research and Business Development foundation, Chonnam National University, 77 Yongbong-ro, Yongbong-dong, Buk-gu, Gwangju, 61186 Republic of Korea; 2 Department of Thoracic and Cardiovascular Surgery, Chonnam National University Hospital and Medical School, 160 Baekseo-ro, Dong-gu, Gwangju, 61469 Republic of Korea; 3 Department of Pediatrics, Chonnam National University Children’s Hospital and Medical School, Gwangju, 61469 Republic of Korea; 4 Department of Mechanical Engineering Graduate School, Chonbuk National University, 567 Baekje-daero, Deokjin-gu, Jeonju, 54896 Republic of Korea; 5 CGBio Co. Ltd., 244 Galmachi-ro, Jungwon-u, Seongnam, 13211 Republic of Korea; 6 School of Mechanical Engineering Chonnam National University, Repubic of, Gwangju, 61469 South Korea

**Keywords:** Vascular graft, Electrospinning, Nanofibers, Medium-term performance, Tissue regeneration, 3D printing, Rat abdominal aorta replacement model

## Abstract

**Background:**

The poor performance of conventional techniques used in cardiovascular disease patients requiring hemodialysis or arterial bypass grafting has prompted tissue engineers to search for clinically appropriate off-the-shelf vascular grafts. Most patients with cardiovascular disease lack suitable autologous tissue because of age or previous surgery. Commercially available vascular grafts with diameters of < 5 mm often fail because of thrombosis and intimal hyperplasia.

**Result:**

Here, we tested tubular biodegradable poly-e-caprolactone/polydioxanone (PCL/PDO) electrospun vascular grafts in a rat model of aortic interposition for up to 12 weeks. The grafts demonstrated excellent patency (100%) confirmed by Doppler Ultrasound, resisted aneurysmal dilation and intimal hyperplasia, and yielded neoarteries largely free of foreign materials. At 12 weeks, the grafts resembled native arteries with confluent endothelium, synchronous pulsation, a contractile smooth muscle layer, and co-expression of various extracellular matrix components (elastin, collagen, and glycosaminoglycan).

**Conclusions:**

The structural and functional properties comparable to native vessels observed in the neoartery indicate their potential application as an alternative for the replacement of damaged small-diameter grafts. This synthetic off-the-shelf device may be suitable for patients without autologous vessels. However, for clinical application of these grafts, long-term studies (> 1.5 years) in large animals with a vasculature similar to humans are needed.

**Supplementary Information:**

The online version contains supplementary material available at 10.1186/s13036-022-00313-9.

## Introduction

Cardiovascular disease is among the leading causes of death worldwide, especially among older adults, and has increased in prevalence since the emergence of coronavirus disease in 2019. The American Heart Association has predicted that by 2035, > 130 million American adults will have some type of heart disease; at present, one in four deaths in the United States is caused by cardiovascular disease [1].

Bypass grafting remains the best treatment for diseased vessels. Although autologous vessels are the gold standard for bypass surgeries, this approach has the drawbacks of limited availability, high morbidity, and complications that may arise after isolation [2–4]. Therefore, there is a need for a safe and efficient vascular repair technique that can be applied in clinical practice. Synthetic vascular grafts have been introduced, including grafts fabricated from expanded polytetrafluoroethylene and polyethylene terephthalate (Dacron); such grafts are suitable for medium- to large-diameter vessels (≥ 6 mm) [5]. Over the past four decades, numerous studies of small-diameter implants used in > 1 million patients reported rapid failure caused by thrombosis, infection, poor somatic growth [6], intimal hyperplasia, compliance mismatch, and other complications [7, 8]. Considerable research has been conducted to improve the outcomes of surgeries using these materials via surface modification and cell seeding approaches. However, the long-term results have been unsatisfactory [9]. Therefore, these prosthetic materials are no longer available for coronary artery replacement or bypass surgeries with diameters (< 6 mm) [10].

Tissue-engineered vascular grafts were introduced to mimic the native vasculature (i.e., collagen and elastin fibers) in terms of structural and biomechanical performance [2]. Such grafts are intriguing because they may reduce complications and enhance growth potential, thus enabling neo-tissue formation from a patient’s cells. However, high processing costs, complex ethical and regulatory issues, uniform cell distribution within scaffolds, quality assurance, and long processing times may be obstacles to the implementation of these grafts [11].

As discussed in our previous review, numerous studies have explored strategies to fabricate vascular grafts (e.g., dip coating, bio-printing, use of natural and hybrid materials, self-assembled cell sheets, and electrospinning) [11, 12]. However, strategies based on nanotechnology and an electrospinning approach may allow for long-term success of vascular surgery because their large surface area and interconnected pores enable the production of three-dimensional (3D) vascular morphologies and structures similar to the natural extracellular matrix. Exploiting the body’s regenerative potential may give rise to a better “bioreactor” than the current in vitro tissue engineering approach because implanted acellular scaffolds undergo significant remodeling after colonization by host cells.

To design an acellular graft that can accelerate host remodeling, we considered the material, porosity, thromboresistance, and reinforcement. In terms of material, we fabricated a two-layer scaffold composed of fast bio-absorbable polymer (polydioxanone (PDO)) on the luminal layer, and a fast bio-absorbable (PDO) and slower absorbable polymer (poly-e-caprolactone [PCL]) were co-electrospun on the abluminal layer. This tailored degradation profile is essential for promoting host remodeling [13]. Concerning porosity, we constructed the luminal layer to be minimally porous, thereby preventing plasma leakage and sweating; the abluminal layer was designed to be highly porous, which facilitates transmural cell colonization of the scaffold. With respect to thromboresistance, we incorporated an antithrombotic drug (dipyridamole) into the fiber matrix; the slow release of this drug inhibits thrombus formation. Finally, we used a 3D printing approach to add reinforcements that prevent aneurysm formation during scaffold degradation.

We achieved good results in short-term studies (2 and 4 weeks) combining reinforced PCL with a PDO vascular graft in a porcine model of carotid artery interposition [12]. This is as a result of good mechanical property of the scaffold such as tensile strength, young modulus and elongation at break (4.12 ± 0.87 MPa, 81.04 ± 3 MPa and 1024 ± 5% respectively) [12]. More so, the reinforcement enhances the cyclic compression strength by 13%. Here, we investigated the medium-term performance of our vascular graft in a rat model of infrarenal abdominal aorta replacement.

## Methods

### Materials and ethical approval

Both PCL (M_n_ = 80,000 g/mol) and PDO pellets were purchased from Sigma Aldrich (USA), while 1, 1, 1, 3, 3, 3-hexafluoro-2-propanol (HFIP) and dipyridamole were purchased from Tokyo Chemical Industry Co., Ltd. (Japan). Chloroform, methanol, and ethyl alcohol were purchased from Samchun Chemical (Korea). Medical-grade biodegradable PCL granules were purchased from ROKIT (South Korea). All animal studies were performed in accordance with the ethical guidelines of Chonnam National University Hospital and Medical School (South Korea). All protocols were approved by the Chonnam National University Animal Ethics Committee.

### Graft fabrication

Vascular grafts were fabricated in accordance with our previously described procedure, with slight modifications [14]. Briefly, PCL was dissolved in a 5:1 ratio of chloroform and ethyl alcohol at 10% (w/v) concentration; PDO pellets were placed in HFIP at 8% (w/v) concentration in a separate vial. To each polymer solution, 10 w/w% of dipyridamole was added and allowed to dissolve over 5 h. The two solutions were separately poured into a 12-mL syringe connected to flexible silicon tubing, which was attached to a programmable pump; the flow rate was 1 mL/h, and the solutions were passed through a 21-G needle. A high-voltage generator (16.1 kV) was applied to the rotating mandrel (1.7-mm Dia). The luminal layer was composed of a thin layer of PDO nanofibers; the abluminal layer was simultaneously spun with PDO and PCL fibers positioned at 80 mm gap apart between the nozzle and the mandrel (Additional file 5: Fig. 4). The mandrel diameter can be adjusted to produce various graft diameters at a rotating speed of 1700 rpm however, for this experiment we used 1.7-mm mandrel. Uniform thickness was obtained as the spinneret moved in a transverse longitudinal direction. The fabricated grafts were transferred to a 3D printer for reinforcement with PCL. The gap between the mandrel and the printing head was adjusted to 0.3 mm, and the printing temperature was set to 90 °C. The 3D-printed coil had a gap of 2 mm and a thickness of 0.16 mm. The resulting grafts were placed in a vacuum at room temperature for 48 h to remove residual HFIP, chloroform, and ethyl alcohol; they were then sterilized with ethylene oxide. Finally, they were cut into lengths of 20 mm and stored at room temperature before use.

### Drug release study

We conducted and in vitro drug release on PCL/PDO nanofibers loaded with three different concentrations of DY 5, 7, and 10% (w/w) with respect to the total polymer weight which was analyzed separately (*n* = 3 each). The PCL and PDO concentration was kept at 10 and 8%(w/v) respectively. The scaffold containing different drug concentrations - 5, 7, and 10%(w/w) DY were denoted as PCL/PDO + 5%DY, PCL/PDO + 7%DY, and PCL/PDO + 10%DY respectively. Pre-weighted nanofibers (≈ 0.5 g) were placed in 5 mL of phosphate-buffered saline (PBS) (pH 7.4) at 37 °C with mild shaking. At designated time points, 1 mL of release medium was pipetted out and kept at − 20 °C, and the volume was replaced with an equal amount of fresh PBS. The dipyridamole concentration in the release medium was measured by UV–vis spectroscopy (SCINCO Mega-800; Scinco Co. Ltd., Seoul, South Korea) at a wavelength of 405 nm. A standard curve was plotted using a series of known dipyridamole contents in 50% ethanol/PBS.

### Implantation

All animal protocols were approved by the Ethics Committee of Chonnam National University Medical School and Chonnam National University Hospital, in accordance with the “Guide for the Care and Use of Laboratory Animals” published by the United States National Institutes of Health (NIH Publication No. 85-23, revised 1996). The experimental animals used in this study were male Lewis rats (weight: 250–300 g, from Samtako Bio Korea Co. Ltd., Korea).

In total, 10 grafts were soaked in heparin solution and successfully implanted into the infrarenal aortas of the experimental rats via an interposition grafting procedure. During surgery, one rat died of excessive bleeding from an injured mesenteric artery and was therefore excluded from the study. The remaining nine rats survived the implantation procedure; all demonstrated patency and showed no clinical signs of failure, according to ultrasonography, throughout the study period. All grafts were explanted at 3 months after implantation. Coagulation therapy was not performed throughout the study period. The surgical survival rate after successful implantation of the 10 reinforced grafts was 92.3% (9/10).

Before interposition graft implantation was performed in the abdominal aorta, each rat was placed in a custom-built chamber supplemented with combined isoflurane and oxygen. The rat was immobilized in the supine position on top of a heating pad (to avoid hypothermia), which was placed on the operating table. For graft implantation, the area was shaved and cleaned with 70% alcohol; a midline incision was created to expose the abdominal aorta and separate it from the inferior vena cava. The infrarenal abdominal aorta was cross-clamped; a 2–3-mm segment was transected and replaced with an 8–10-mm PCL/PDO scaffold. The graft and native aorta were connected by end-to-end anastomosis with 10-0 proline sutures. After surgery, the rats were housed in temperature-controlled rooms with a 12 – h light-dark cycle, with standard food and water available. Rats were not administered anticoagulant or antiplatelet treatment before or after surgery. All grafts (*n* = 9) were explanted at 12 weeks post-implantation.

### Angiography

Rats (*n* = 5) were anesthetized with isoflurane (1–2.5%) and administered intraperitoneal ketamine to alleviate post-procedure discomfort. A 4-French sheath was inserted into the exposed rat carotid artery. Abdominal aortic angiography was conducted using fluoroscopic guidance and a mobile fluoroscopy system (BV Pulsera; Philips Medical Systems, USA). Based on the angiography findings, the nonionic contrast agent Omnihexol 300 (Korea United Pharm Co., South Korea) was used.

### Ultrasound observation

Flow through the graft was evaluated postoperatively on the rats (*n* = 5) at 12 weeks after they were anesthetized and placed in a supine position with their limbs restricted. At 12 weeks transabdominal Doppler was performed using Vivid S5(GE Medical system, 4 Etgar street 39,120 Tirat Carmel, Israel) with i12L-RS probe. The graft patency, diameter and mid-graft flow pattern was measured.

### Histological analysis

Samples were sectioned (8 μm thickness) and deparaffinized by three incubations in xylene (5 min each), and then hydrated in descending concentrations of alcohol (99.9, 95, and 70%) and subsequently washed in distilled water. H&E, Masson’s trichrome, Safranin O, Verhoeff-Van Gieson, and Orcein staining for elastic fibers were performed in accordance with the manufacturer’s protocols. Subsequently, the samples were dehydrated and mounted on slides for observation via optical microscopy.

### Lumen area, wall thickness and fluorescence intensity measurements

To quantify the lumen area and graft wall thickness, 8-μm frozen cross-sections from three different 12-week neografts were stained with hematoxylin and eosin (H&E). Control samples were native abdominal aorta (*n* = 3 × 3 sections) from a healthy rat of the same age. All samples were processed identically, and images were examined using Image. J software (NIH, USA). Briefly, after uploading the image on the software and setting up the scale. The image converted to 8 bit followed by clicks on process> binary> make binary. Go to analysis> set measurement and check area. Set the light headed tool and click on the lumen area. A yellow boundary will be seen only in the lumen. Then go to analysis > click measure (Ctrl+M) and save the result in excel.

For mean fluorescence measurement, Immunofluorescence image was uploaded in the program and a scale was set. Go to image>color> split channel and work with color of interest. Go to analyze>set measurement and check mean gray value and area. To record macros, go to plugin>macros and record. Choose size and shape from area icon (e.g square). Click create on pop up menu>file>save as with file in. ijm > save. Finally, tell the program to analyze and measure.

### Immunofluorescence staining

Briefly, paraffin was removed from 8-μm explant sections by three incubations in xylene (5 min each), followed by hydration with descending concentrations of alcohol (99.9, 95, and 70%) and washing with distilled water. Next, the samples were transferred to 3% hydrogen peroxide for 10 min and washed three times with phosphate-buffered saline (5 min each). Antigen retrieval was conducted using citrate buffer (pH 6.0) for 10 min at 97 °C. Samples were blocked with 1% bovine serum albumin in phosphate-buffered saline for 1 hr. and then washed three times with phosphate-buffered saline (5 min per wash). α-Smooth muscle actin (SMA) staining was performed using mouse anti-human α-SMA primary antibody (mca5781ga; Bio-Rad). Myosin heavy chain staining was performed using an anti-cardiac myosin heavy chain primary antibody (Invitrogen, USA) and AlexaFluor 597 goat anti-mouse secondary antibody (Invitrogen). Endothelial cell staining was conducted using fluorescein isothiocyanate-linked anti-von Willebrand factor (vWF) primary antibody (mbs2016029; [MyBioSource, USA]) and AlexaFluor 488 goat anti-rabbit secondary antibody (Invitrogen). Collagen I/III staining was carried out using rabbit anti-pig collagen I/III polyclonal antibody (Bio-Rad). Newly recruited macrophages were visualized using an anti-CD68 primary antibody (Invitrogen) and AlexaFluor 597 goat anti-mouse secondary antibody (Invitrogen). The dilution factor for both primary and secondary antibodies was 1:100 dilutions. Nuclei were stained by incubation with 4′,6-diamidino-2-phenylindole (DAPI). All antibodies were used in accordance with the manufacturer’s protocols. Samples were washed three times with phosphate-buffered saline (5 min each) and then coverslipped with mounting media. Fluorescence images were captured by laser scanning confocal microscopy. Native pig carotid artery sections were used as positive controls.

### Scanning electron microscopy

As fabricated electrospun samples which was cross-sectioned and prepared in 2D form (1 cm X 1 cm) as well (*n* = 5 each group) were vacuum dried and sputter-coated with a thin layer of gold and observed under a scanning electron microscope (EM-30AX; COXEM Co., Ltd., South Korea) with a 15 kV medium beam to visualize surface morphology, thickness and the attachment of the 3D reinforcement.

In-vivo sample: Immediately after tissue explantation (n = 5), the lumen of each neovessel was flushed with heparinized saline and placed in 100% formalin. Two days before observation by microscopy, the samples were transferred into glutaraldehyde and incubated overnight. The samples were rinsed, washed three times with fresh buffer (without fixative), and dried in a graded alcohol series (e.g., 50–100%) for 15–20 min per alcohol solution. The 100% alcohol step was repeated and samples were placed into a 1:2 solution of hexamethylenedisilane: ethanol for 20 min. Subsequently, they were transferred to a 2:1 solution of hexamethylenedisilane: ethanol solution for 20 min. Finally, the samples were transferred into 100% hexamethylenedisilane solution; they were incubated at room temperature overnight in a fume hood, with the container cap loosely closed. Samples were sputter-coated with a thin layer of gold and observed under SEM with a 15 kV medium beam to visualize native vessel morphology, neotissue formation, and endothelialization.

### Statistical analysis

Unless stated otherwise, all data are presented as the mean of at least three independent experiments. SEM imaging of neo-artery at end-point(*n* = 5) was performed with five independent samples as replicate and these samples was divided cross-sectional for Immunofluorescence staining(n = 5). For H&E staining, four independent samples (*n* = 4) as replicate was used at end-point. Error bars on all graphs represent standard deviations of the mean based on the total number of samples. One-way analysis of variance (ANOVA) was used to determine the significant differences and any reference to a difference in the Results and Discussion section implies statistical significance at the level *P* < 0.05. For microscopy imaging, at least four regions were analyzed for each experiment.

## Results

**Fig. 1:**
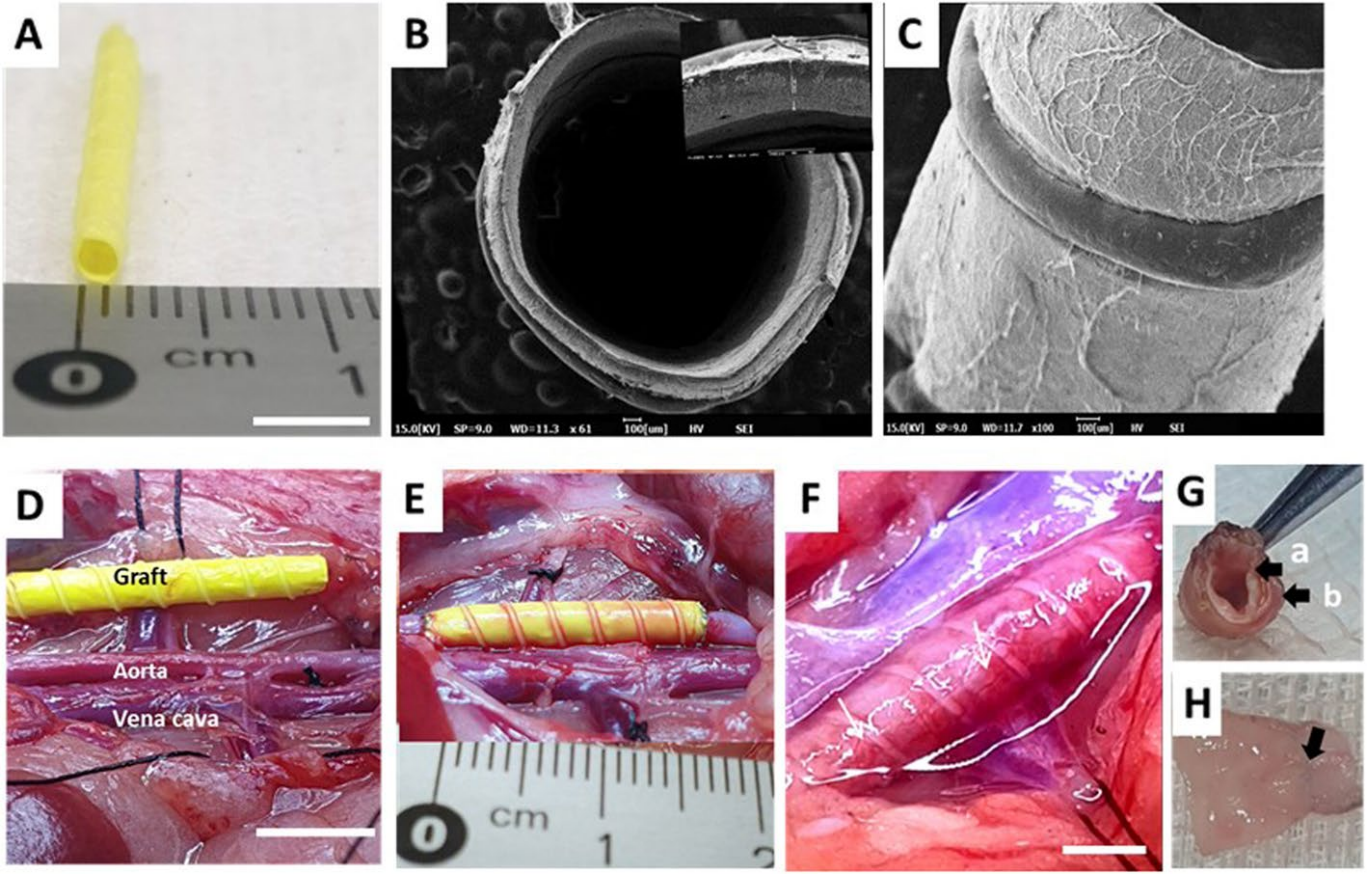
Structural characterization of small-diameter grafts, surgical procedure overview, and remodeling at 3 months post-implantation. **A** As-fabricated PCL/PDO grafts have an internal diameter of ~ 1.7 mm and length of 15 mm. **B** Cross-sectional scanning electron microscope images shows wall thickness of ~ 100 μm (magnified in the inset). **C** Scanning electron microscope image shows a side view of graft with 3D reinforcement and a microfibrous outer layer. **D** Gross image of isolated infrarenal abdominal aorta and corresponding PCL/PDO graft prior to implantation. **E** Gross surgical view of PCL/PDO graft anastomosis. The rat’s head is situated beyond the upper left quadrant of the image. **F** Graft remodeling at 3 months post-implantation. White arrows indicate the formation of tiny visible blood vessels on the engineered graft. Scale bar, 5 mm. **G** A 1.7-mm graft explanted from a rat at 3 months post-implantation demonstrates the formation of endothelium (black arrow, a) and an adventitial layer (black arrow, b). **H** A 1.7-mm PCL/PDO graft was explanted from the rat aorta at 3 months post-implantation (arrow indicates anastomotic suture line)

Biodegradable vascular grafts (*n* = 10) were engineered from PCL and PDO-based materials using an electrospinning approach illustrated in Additional file 5: Fig. 4A and then subjected to scanning electron microscopy for characterization of the inner diameter, wall thickness, and luminal/abluminal surface topologies (Fig. 1A–C). Both luminal and abluminal surfaces exhibited a microporous morphology indicative of cell infiltration into the PCL/PDO graft (Additional file 5: Fig. 4A&C respectively), as we previously reported [12]. The pore area was high in the outer layer as compared to luminal surface (137.9 ± 4.2 against 70.1 ± 4.9 μm^2^ respectively; Additional file 5: Fig. 4D). A high pore interconnectivity is important for efficient cell infiltration, which is the first step in the initiation of host remodeling. The dipyridamole loaded in the fibers is expected to make the lumen anti-thrombogenic and inhibit blood clotting. After implantation into rat infrarenal abdominal aortas without systemic heparin treatment, significant remodeling was observed (Fig. 1D–H). There was no observable constructive fibrotic tissue around the grafts prior to explantation (Fig. 1F), instead, the grafts were covered with fascia that resembled that of a native aorta. Figure 1G shows a gross image of a graft prior to explantation. White arrows indicate well-formed blood vessels; reinforcement is obvious at this time point and functions to prevent aneurysmal dilation while the nanofiber degrades. This finding is consistent with our hypothesis that the remodeled grafts are compositionally similar to native vessels, although the differences were minimal at this time point (Additional file 1: Supplementary Video 1). Gross analysis of explanted grafts revealed a smooth luminal surface with an adventitial tissue layer (Fig. 1G). The grafts exhibited good integration with the native vasculature at anastomotic sites; there were no signs of unusual tissue adhesions, perigraft hematomas, seromas, or inflammatory reactions (Fig. 1H). Additionally, the luminal surface was shiny, with no evidence of stenosis or aneurysm on the explanted grafts (Fig. 1H; Additional file 3: Supplementary Video 3). Macroscopic observation revealed a completely endothelialized surface.

We investigated 3 different concentration of dipyridamole release pattern embedded in the graft material over 35 hours (Additional file 5: Fig. 4E). The cumulative release of the samples reached a plateau after 10 hours, following a slow release over the experimental window (275 hours). As expected, a slower release was observed for PCL/PDO + 10%DY compared to the PCL/PDO + 5%DY scaffold over the whole release process. At 10 hours 12.3, 6.7, and 0.9% of the drug were released from PCL/PDO + 5%DY, PCL/PDO + 7%DY, and PCL/PDO + 10%DY drug-loaded scaffolds, respectively. Sustained release of drug from the PCL/PDO + 10%DY drug-loaded scaffold could be suitable for inhibiting platelet activation on the implanted graft surface over a long period, thus we choose this sample for the in-vivo experiment.

### Digital subtraction angiography and ultrasonography findings

**Fig. 2:**
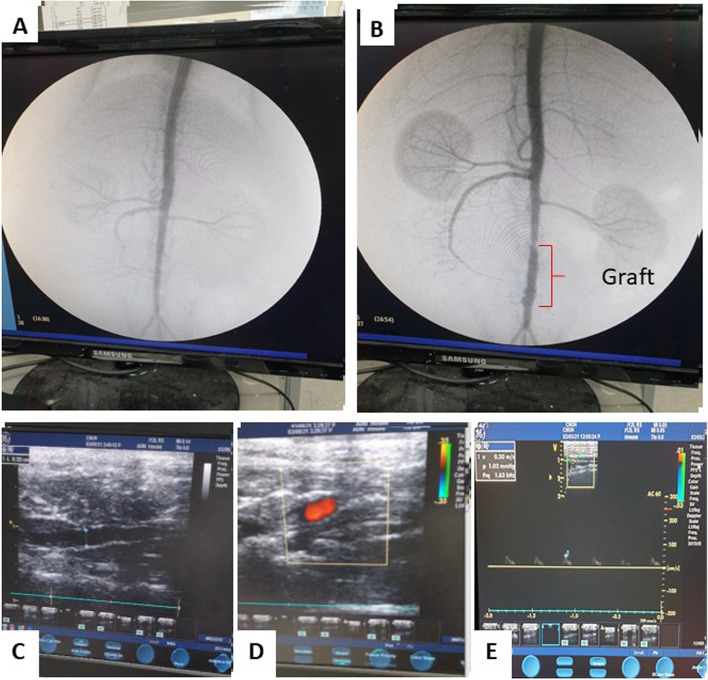
Monoplanar (anteroposterior) projection images of digital subtraction angiography at 12 weeks after implantation (*n* = 3). **A** Normal rat abdominal aorta (control). **B** PCL/PDO graft indicated by red lines. **C** B-mode ultrasonography with a patent PCL/PDO graft indicated by the blue dotted line. **D** Color Doppler showing blood flow through the graft. **E** Pulse Doppler

Standard microsurgical techniques were employed to implant PCL/PDO grafts into rat aortas; all grafts were implanted by one surgeon (MJ). At 12 weeks post-implantation, the grafts exhibited good patency; no thrombi were observed in the lumen when assessed by angiography and Doppler ultrasonography (Fig. 2A–C). The Doppler images showed a lumen diameter of 2 mm (Fig. 2C), which is typical diameter of a native rat aorta. At 12 weeks after implantation, angiography assessment of the grafts revealed no obvious stenosis or dilation (Fig. 2B). Additionally, dynamic Doppler ultrasonography showed that the graft exhibited good compliance and pulsed spontaneously with the native aorta (Fig. 2C–E; Additional file 2: Supplementary Video 2).

### Histology analysis

**Fig. 3:**
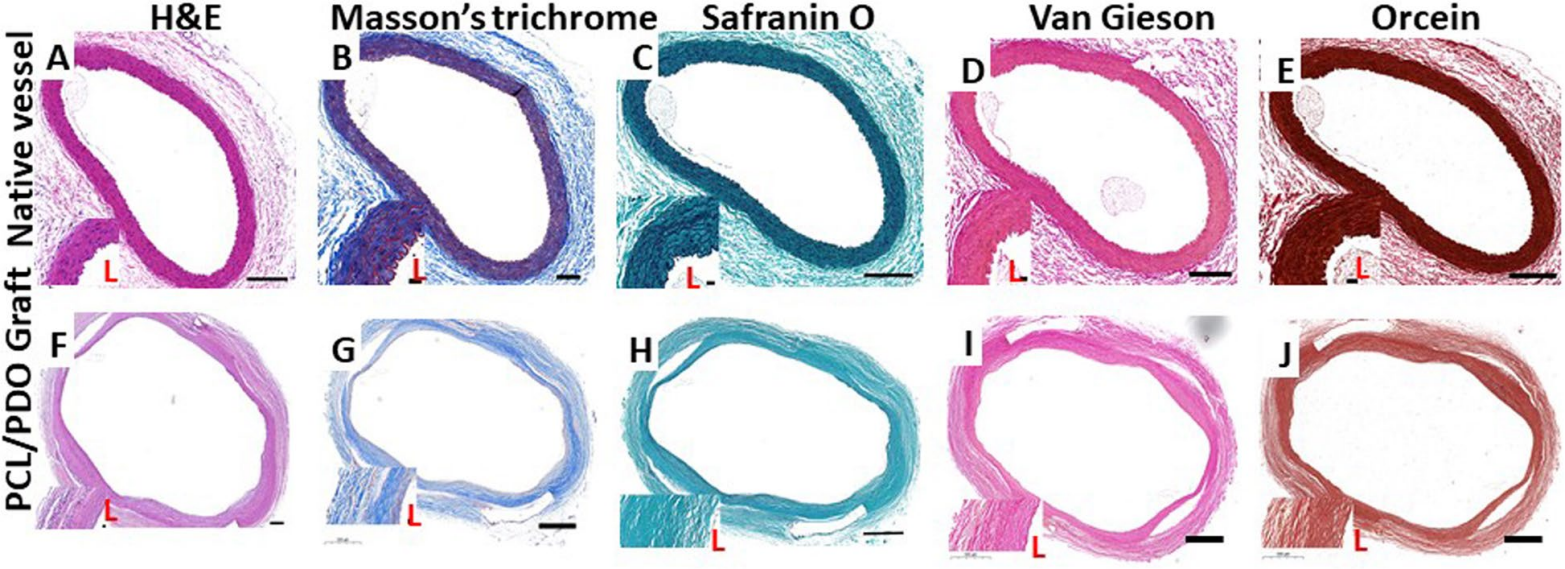
Remodeling of the PCL/PDO graft at 12 weeks after implantation compared with native aorta. Top and bottom rows show the native vessel and PCL/PDO graft, respectively. **A** and **F** H&E staining. **B** and **G** Masson’s trichrome staining reveals collagen in the explanted graft and native aorta. **C** and **H** Safranin O staining. **D** and **I** Verhoeff-Van Gieson staining. **E** and **J** Orcein staining for Elastic fibers. Scale bars: top row, 200 μm (inset, 20 μm); bottom row, 500 μm (inset, 20 μm). L = lumen

We compared the results of cross-sectional histological staining between native rat aorta and PCL/PDO grafts after explantation at 12 weeks (Fig. 3). H&E staining of native rat aorta showed continuous red tissue and a typical round blood vessel shape, similar to PCL/PDO grafts (Fig. 3A and F). However, a thin grayish-white layer was present inside the PCL/PDO graft, which represented polymer remnants. In PCL/PDO grafts, the layered structure was clearly preserved with considerable host cell infiltration and secretion of substantial extracellular matrix between the layers. The inner lumen of the PCL/PDO graft was composed of an integrated layer of cells with abundant extracellular matrix deposition, which suggested that the graft could support host cell migration and proliferation. These H&E staining results demonstrate the excellent performance of PCL/PDO grafts in maintaining tubular shape and a thrombus-free surface at 12 weeks. Thus, the results confirm appropriate remodeling by PCL/PDO grafts.

To assess the components of extracellular matrix deposited in both native and PCL/PDO grafts, we performed Masson’s trichrome, Safranin O, and Verhoeff-Van Gieson staining (Fig. 3B–J). Masson’s trichrome staining was used to identify muscle fibers and keratin (red) or collagen (blue), Safranin O was used to assess the distribution of proteoglycan [15], and Verhoeff-Van Gieson was used to reveal the presence of elastin fibers. Abundant collagen was observed, which may have been secreted by fibroblasts/smooth muscle cells that migrated into the graft. Cross-sectional staining of PCL/PDO grafts with Masson’s trichrome, Safranin O, and Verhoeff-Van Gieson confirmed that collagen, glycosaminoglycan, and elastin, respectively, were distributed within the interstitium of the graft. The elastin quantity is expected to significantly increase during long-term in vivo studies, in which the duration is sufficient for most polymer fibers to degrade.

The Orcein staining was a traditional method used in staining elastic fibers. Hence, Fig. E&J confirm the presence of elastic fiber however, at this stage the fibers are not fully developed as they still lack the hills and valley shape observed in native aorta.

### Results of immunohistological staining

**Fig. 4:**
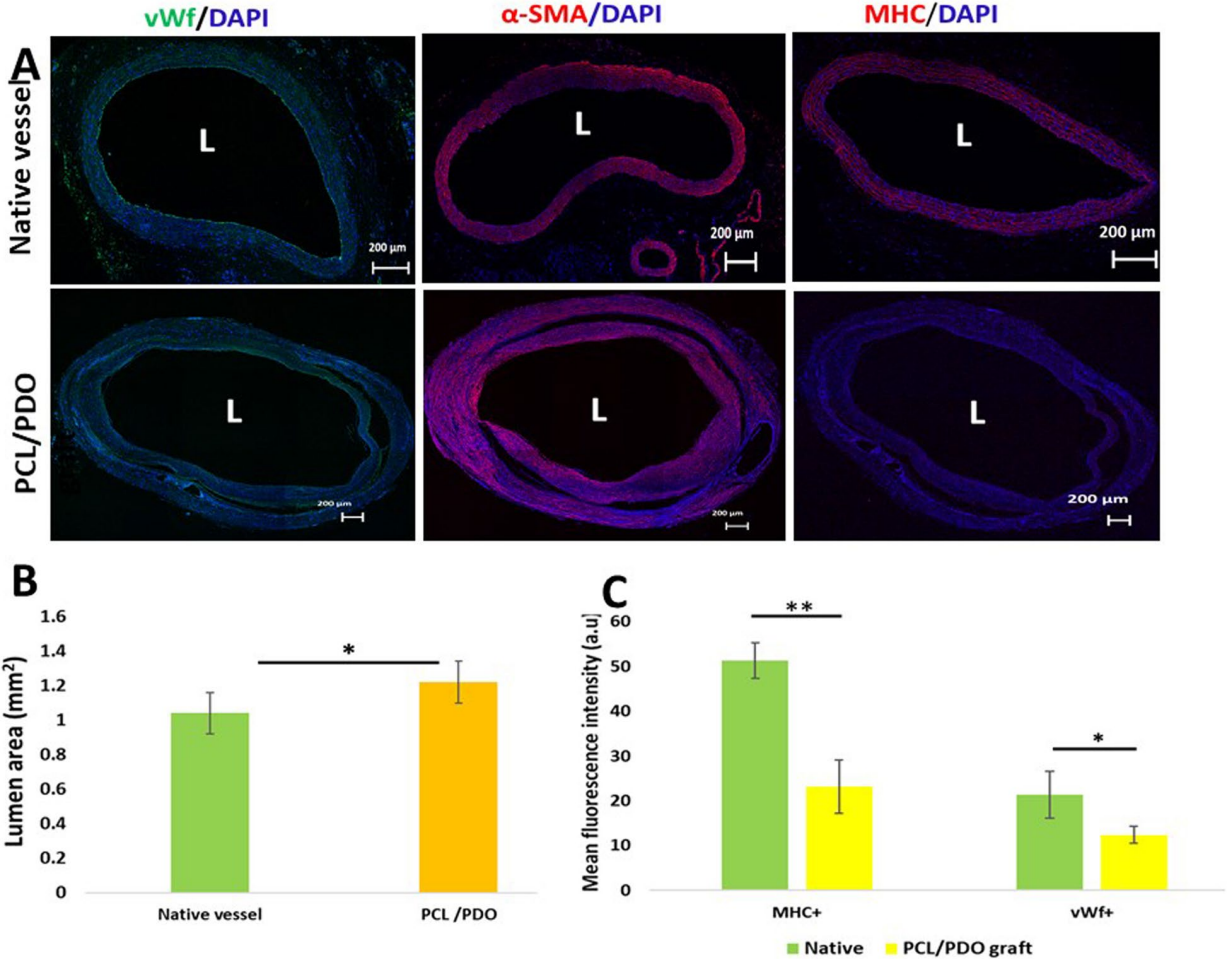
Tissue remodeling and extracellular matrix deposition in explanted grafts at 3 months compared with the native aorta. **A** Cross-sectional images of native vessels and regenerated grafts (top and bottom rows, respectively) were immunostained to examine endothelial cells (green), smooth muscle cells (red), and the distribution of contractile smooth muscles (myosin heavy chain [red]). Endothelial cells were stained with anti-vWF antibody (first column; green, DAPI is stained blue; *P* = 0.007). Smooth muscle cells were stained with anti-α-SMA antibody. **B** L = Lumen area (± standard deviation) of PCL/PDO grafts measured from histological images in millimeters (*P* = 0.01). **C** Mean fluorescence intensity of vWF-positive cells measured from immunofluorescence images. Mean fluorescence intensity of myosin heavy chain (MHC)-positive cells measured from immunofluorescence images (*P* = 0.0026). Data are mean ± standard deviation. *P < 0.05; *n* = 5

We conducted immunofluorescence assays (Fig. 4A) to further explore remodeling within the PCL/PDO grafts. vWF staining revealed a confluent thin layer of endothelial cells covering the lumen (Fig. 4, left column; Additional file 5: Fig. 1). As in previous studies, endothelial cells may have migrated directly from anastomosed vascular tissue, transmurally from surrounding tissue [16], or gradually from circulating progenitor cells [17]. Cells positive for α-SMA (a protein specific to mural cells, including smooth muscle) were organized into circumferential layers within the graft (Fig. 4A, middle column; Additional file 5: Fig. 1A, middle column), which indicated that abundant smooth muscle cells had infiltrated into the graft. There were no signs of neointima or intimal hyperplasia in the explanted grafts. The visible bands of dark lines near the lumen within the interstitium of each graft were presumed to comprise remnants of polymer materials.

The amount of myosin heavy chain expression and specific heavy chain isoforms are the most obvious markers of fully differentiated smooth muscle cells [18]. The weak expression of myosin heavy chain indicated an early developmental stage of neoartery contractile smooth muscle (Fig. 4A, right column; Additional file 5: Fig. 1A). Closer observation indicated that the layers were distinct, with a trilaminar structure similar to that within muscular arteries. Immunofluorescence staining confirmed abundant expression of α-SMA (Additional file 5: Fig. 1, middle) and low expression of myosin heavy chain compared with native vessels. Analysis of the lumen area revealed extensive remodeling, which remained active because the remnant polymer thickness was around 51 μm (yellow arrow) at this time point (Fig. 4B, *P* = 0.01; Additional file 5: Fig. 2). Furthermore, is should be noted that native vessels inherently exhibit vasoconstriction upon isolation because of changes in temperature, functional muscle, and elastic fibers. Mean fluorescence intensity assessment (Fig. 4C) indicated that the number of vWF^**+**^ cells was higher in native vessels than PCL/PDO grafts (*P* = 0.047). Similar findings were observed for myosin heavy chain-positive cells (*P* = 0.0026).

**Fig. 5:**
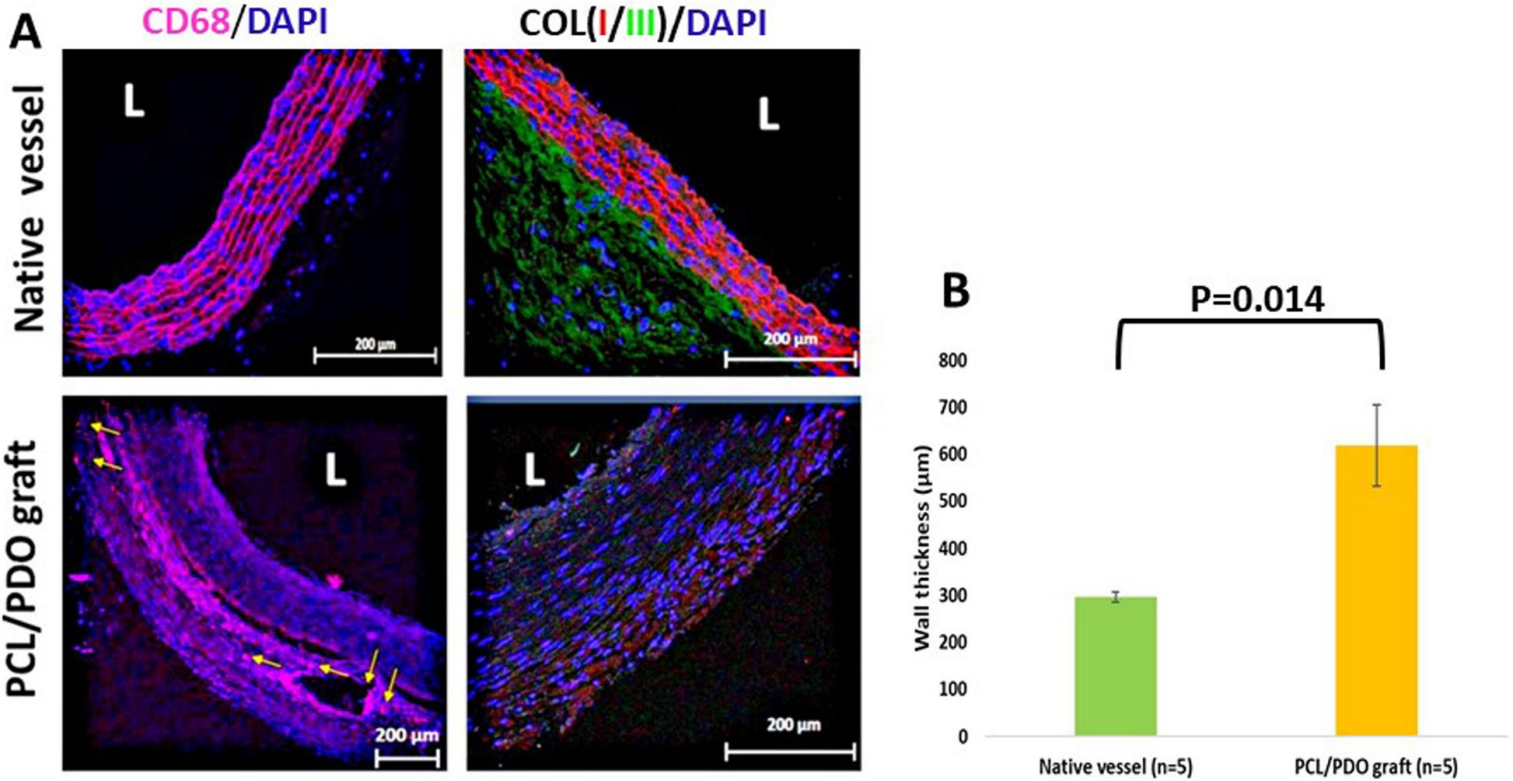
**A** Representative fluorescence micrographs of immunostaining for CD68 (pan-macrophage marker) at 3 months, indicated by the yellow arrows (left column). CD68 was stained red, and nuclei were counterstained with DAPI (blue). Immunostaining of collagen I/III (right column). Collagen type I was stained green, collagen type III was stained red, and cell nuclei were counterstained with DAPI (blue). Images are representative of at least three independent samples. L = lumen. **B** Wall thickness differed between native vessels and PCL/PDO grafts at 3 months

Tissue regeneration and vascular hemostasis are dependent on macrophage polarization. A previous study showed that the presence of CD68-positive cells in the media and adventitia was not correlated with an unfavorable prognosis. However, the presence of CD68-positive cells in the tunica intima was correlated with a higher number of stenosis compared with grafts that lacked such cells [19]. CD68-positive cells (macrophages) were observed in the PCL/PDO grafts (Fig. 5A, first panel), but were mainly localized in the graft remnants (tunica media and adventitia; yellow arrow) at low density, suggesting a minimal inflammatory response and active polymer resorption [20]. This result is consistent with previous findings of a considerable reduction in the number of CD68-positive cells at 4 months after implantation [21]. Additionally, macrophage infiltration is considered an essential step in the transition of tissue-engineered vascular grafts from scaffolds to neovessels [22]. However, prolonged macrophage presence indicates an undesirable chronic inflammatory response [19, 23]. More so, a previous study suggested that the presence of CD68-positive cells, but not foam cells, in the intima of saphenous vein grafts could be an early sign of graft occlusion.

We investigated the extracellular matrix components collagen I and III, which are the most abundant types of collagen participating in the maintenance of vascular structural and functional integrity (Fig. 5A, right column). Collagen I was abundantly distributed throughout the graft area (from the lumen to the adventitial layer), whereas collagen III expression was minimal. This may be the result of collagen III gradually shifting to collagen I during maturation, as seen in previous studies [15, 20]. The wall thicknesses of native vessels and PCL/PDO grafts were 296 ± 10 and 618 ± 87 μm, respectively (*p* = 0.014; Fig. 5B).

### Endothelial regeneration and synchronous pulsation at 12 weeks

**Fig. 6:**
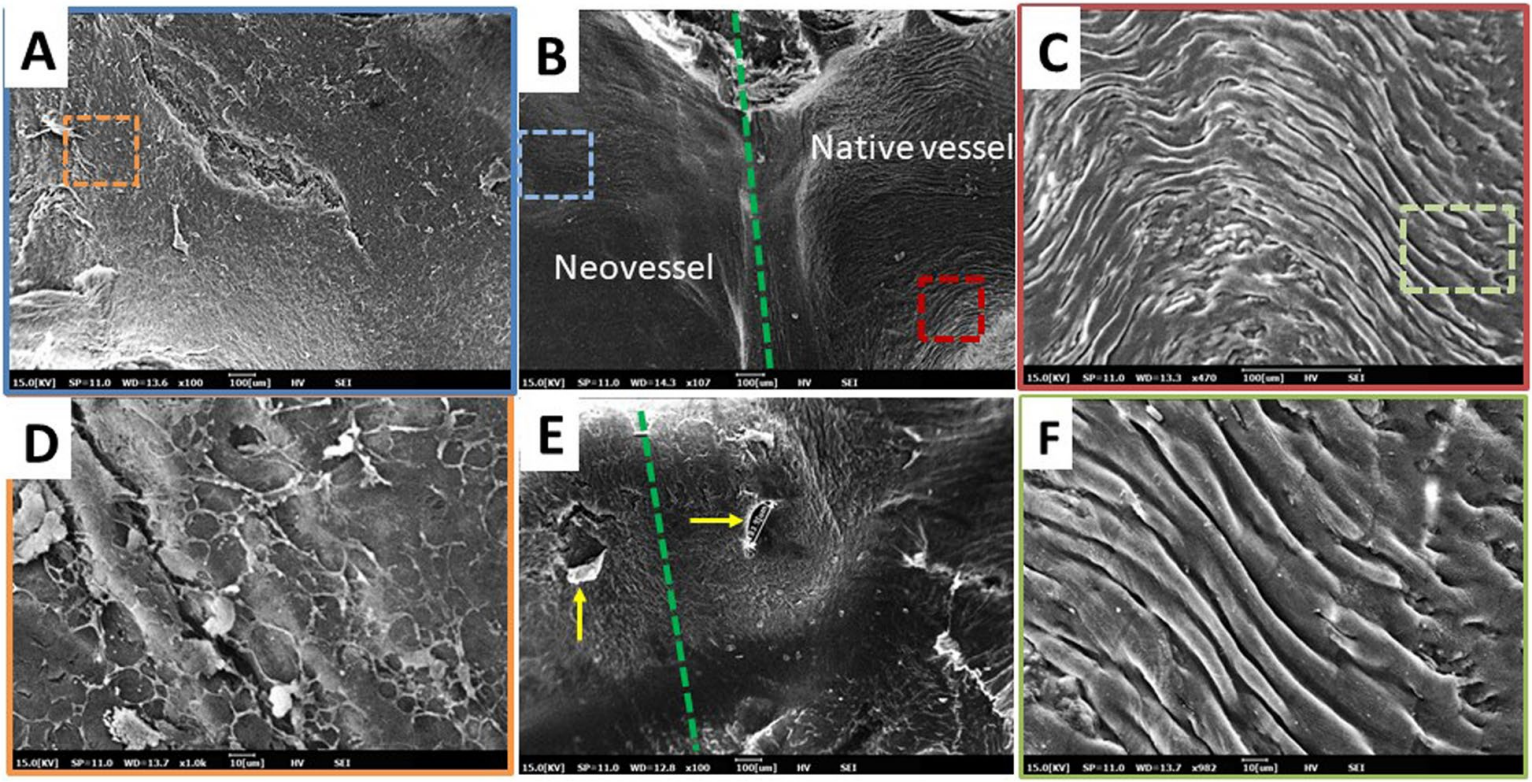
Scanning electron micrographs of the luminal surface of explanted PCL/PDO grafts at 12 weeks compared with native vessels. **A** Representative image of a neovessel (PCL/PDO graft). **B** Anastomotic site; the green dashed line represents the site of anastomosis. **C** Native vessel. **D** High magnification of the area within the brown dashed-line box in **A**. **E** Magnified portion of the anastomotic site, with suture holes indicated by yellow arrows; green line indicates anastomotic site and the transition from native vessel to neovessel). **F** Magnified image of autogenous rat aortic arch wall (green dashed-line box in **C**)

The rate of endothelialization after a vascular injury has important effects on intimal hyperplasia, stenosis, and thrombus formation. Thus, we used scanning electron microscopy to study the graft endothelium at 12 weeks. The endothelial surfaces of the neovessels and native vessels showed similar confluent endothelial cell coverage, without signs of thrombus (Fig. 6A–C). Magnified images of neovessels (Fig. 6D) indicated hills and valleys typical of native vessels, which were not fully developed at this time point. However, there was a dense periendothelial or self-assembled network of endothelial cells on the surface. A previous study suggested that this basket-like network of stellate cells could provide vessels with the capacitance [24] necessary to withstand the pressure arising during remnant polymer degradation. A magnified view of the anastomotic site (Fig. 6E) showed that the needle hole, indicated by a yellow arrow, was preserved; it measured 108.9 μm. The typical luminal surface (covering the intima) of a normal rat aorta is lined by a confluent sheath of elongated and tightly packed endothelial cells that prevents the adhesion of blood cells and other molecules (Fig. 6F). Graft integration with the host tissue remains a major challenge in tissue engineering. However, at 12 weeks our graft exhibited great patency with synchronous pulsation and resembled the host aorta (Additional file 1: Supplementary Videos 1, Additional file 2: Supplementary Video 2 and Additional file 4: Supplementary Video 4). Consistent and clear pulsation of the neoartery indicated excellent integration with the host tissue; this integration corresponds to a confluent endothelium that smoothly transitions from native vessel to neoartery (Fig. 6B). The transitions indicated by the white dotted lines at the anastomotic site indicate abundant collagen deposition in neovessels (Additional file 5: Fig. 3A and B).

## Discussion

Over the past 2 decades, there has been considerable research on biodegradable materials for medical applications because of their intriguing properties [25, 26]. In the field of tissue engineering, commercially available biostable materials (e.g., expanded polytetrafluoroethylene and polyethylene terephthalate) are slowly being replaced with biodegradable materials because the prolonged presence of biostable materials causes tissue stiffening due to fibrous encapsulation; this may activate inflammatory cells and cause intimal hyperplasia [27]. Among the biodegradable polymers, micro/nanofibers of PCL, PDO, polylactic acid, and polyglycolic acid have received particular attention from tissue engineers because of their biocompatibility, mechanical properties, and adjustable biodegradation time. These properties are of considerable interest for the medical and pharmaceutical industries. Additionally, electrospun nanofibers and microfibers made from biodegradable polymers could serve as vascular prostheses because their high porosity, small fiber size, small pores, and large surface area provide more space for cell infiltration, proliferation, and matrix production [28, 29]. However, electrospun grafts are more likely to experience postoperative mechanical failure (aneurysms) because of high arterial pressure.

Previous investigations revealed significant aneurysmal dilation caused by premature loss of strength when PDO was used alone to fabricate vascular grafts [30]; premature loss of strength is a major cause of failure involving fast degradable polymers [31]. Conversely, despite the many favorable characteristics of PCL vascular prostheses, chondroid metaplasia-induced calcification develops after 6 weeks [32]; this remains a major limitation of PCL grafts. Previous studies of polylactide–polyglycolide grafts showed good patency in both canine and porcine models [33–35]; however, slow graft degradation limited cell infiltration and prolonged the presence of foreign materials. Additionally, the extracellular matrix content has not been fully described in the literature. Our 1-month comparative analysis of PCL/PDO grafts with expanded polytetrafluoroethylene grafts in a porcine carotid model showed high remodeling potential, which motivated the present study.

Here, we successfully conducted a medium-term study (12 weeks) of reinforced 1.7-mm grafts in a rat model of aortic interposition, which showed rapid remodeling. The two-layer grafts were fabricated through a combination of electrospinning and 3D printing, similar to the approach used in our previous study with slight modification. The 3D printing allowed external reinforcement of the tubular nanofiber construct with thin PCL threads. The use of thin, biodegradable PDO nanofibers as the luminal layer minimized the effect of larger fibers, which could disrupt laminar blood flow and inhibit endothelial cell attachment. Moreover, large fibers can promote platelet adhesion and activation [36, 37]. The grafts showed nearly complete host remodeling at 12 weeks; at the conclusion of the study, graft patency was assessed by noninvasive ultrasound techniques [38] and angiography [39]. To reduce the number of anesthesia procedures, the rats were examined only on the days of implantation and sacrifice. The grafts resisted intimal hyperplasia and had 100% patency, as determined by angiography. Immunostaining revealed significant infiltration of α-SMA-positive cells, endothelial cell coverage of the luminal surface, the presence of collagen I and III, and low numbers of inflammatory cells (CD68), suggesting that the device has regenerative potential. It is difficult to determine the sources of the observed endothelial and α-SMA-positive cells. However, they may have migrated transmurally, differentiated from endothelial progenitor cells, or migrated directly from native vessels at anastomotic sites according to previous studies [40–42]. The conversion of vascular smooth muscle cells into endothelial cells through mesenchymal endothelial transition also cannot be excluded, although further investigation is required. Notably, the presence of elastin in the explanted graft wall appears to have an important role in aortic reconstruction. The wall thickness was greater in PCL/PDO grafts than native vessels (600 ± 100 μm against 290 ± 10 μm; Fig. 5B). This may be related to the presence of polymer nanofiber remnants, because extracellular matrix proteins are not fully developed at this time point; therefore, the wall thickness is expected to decrease during the course of long-term studies, in which all polymers are degraded.

Endothelialization and good mechanical strength are the most important determinants of long-term graft survival. Our graft prevented thrombosis and allowed endothelial cells to form a monolayer that contributed to the observed medium-term patency. Both immunofluorescence staining and scanning electron microscope analyses of explants revealed excellent endothelialization of our grafts. Histological analysis and pre-isolation graft images indicated that the presence of 3D-printed PCL reinforcement at 12 weeks significantly enhanced mechanical strength by inhibiting aneurysm formation during polymer degradation (PDO fibers).

The grafts were closely integrated with the native vasculature, as indicated by angiogenesis and smooth muscle cell recruitment in the graft interstitial space. Prior to explantation, graft porosity-induced neocapillary formation was observed; this may be attributed to the porous nature of PCL/PDO grafts, as demonstrated in a previous study [43]. The presence of a minimal inflammatory response, as demonstrated by the low amount of CD68-positive cells, suggests that PCL/PDO electrospun fibers are suitable biocompatible materials for vascular regeneration.

## Conclusions

Electrospinning offers a more flexible approach to vascular graft fabrication, allowing for scaled-up production of higher-quality and more uniform grafts.

In this study, the regenerative potential of electrospun reinforced porous vascular grafts was explored in a rat model; at 12 weeks, the grafts showed good patency and substantial remodeling. The grafts were highly cellularized, including α-SMA-positive cells, and the luminal surface was completely covered by endothelial cells. Consistent with our hypothesis, the excellent mechanical strength observed at 12 weeks before explantation resulted from spiral 3D reinforcement, where substantial fiber degradation was observed at this time point. Additionally, the grafts showed structural and functional properties comparable to native vessels, indicating their potential application for the construction of tissue-engineered small-diameter vascular grafts.

### Study limitations

We studied biodegradable reinforced grafts in a rat model of aortic interposition for up to 12 weeks. This medium-term study was designed to confirm the potential utility of this graft for bypass surgery and small vessel reconstruction. Although the graft diameter is insufficient for human use, we provide a high-throughput model for testing graft material.

Further studies will be conducted in a large animal model (6 to 1.5 years) to investigate long-term performance, temporal degradation, aneurysmal dilation potential, neo-intimal hyperplasia characteristics, and graft remodeling.

## Supplementary Information

**Additional file 1.** 

**Additional file 2.** 

**Additional file 3.** 

**Additional file 4.** 

**Additional file 5:** **Figure 1.** Tissue remodeling and extracellular matrix deposition in the explanted graft at 3 months as compared to native aorta. Cross-sectional images of native vessel and regenerated grafts (top row and bottom row respectively) were immunostained to examine the endothelial cells, smooth muscle cells, and myosin heavy chain. Endothelial cells were stained with vWF antibody (1st column; green and Dapi stained blue). Smooth muscle cells were stained with α–smooth muscle antibody. **Figure 2.** H&E staining of PCL/PDO graft at 12 weeks indicating remnants of PCL/PDO in the neo-vessel- yellow dotted arrow (51 μm). scale bar = 100 μm. **Figure 3.** Longitudinal section of collagen stained PCL/PDO graft explant at 12 weeks stained. a) Collagen I & III staining was counterstained with Dapi-merge. b) Collagen I&III staining alone. White dotted line indicates anastomosis site. **Figure 4.** (a) Fabrication process of graft. (b&c) Morphology of the luminal and outer surface of the scaffold respectively. (d) Pore area measurement using image. J (p = 0.0001). (e) In vitro cumulative drug release of dipyridamole from nanofibers containing various concentrations of drug (PCL/PDO + 5%DY, PCL/PDO + 7%DY, and PCL/PDO + 10%DY). The insert shows a higher magnification view of the area indicated with a red dotted circle.

## Data Availability

All relevant data will be freely available post-publication to any scientist that show interest or made a request.
