# Supplementary material for: Rapid remodeling observed at mid-term in-vivo study of a smart reinforced acellular vascular graft implanted on a rat model
Source: J Biol Eng. 2023 Jan 3;17:1. doi: 10.1186/s13036-022-00313-9 (PMC9810246; doi:10.1186/s13036-022-00313-9)
Supplement: Supplementary file 5 — Additional file 5: Figure 1. Tissue remodeling and extracellular matrix deposition in the explanted graft at 3 months as compared to native aorta. Cross-sectional images of native vessel and regenerated grafts (top row and bottom row respectively) were immunostained to examine the endothelial cells, smooth muscle cells, and myosin heavy chain. Endothelial cells were stained with vWF antibody (1st column; green and Dapi stained blue). Smooth muscle cells were stained with α–smooth muscle antibody. Figure 2. H&E staining of PCL/PDO graft at 12 weeks indicating remnants of PCL/PDO in the neo-vessel- yellow dotted arrow (51 μm). scale bar = 100 μm. Figure 3. Longitudinal section of collagen stained PCL/PDO graft explant at 12 weeks stained. a) Collagen I & III staining was counterstained with Dapi-merge. b) Collagen I&III staining alone. White dotted line indicates anastomosis site. Figure 4. (a) Fabrication process of graft. (b&c) Morphology of the luminal and outer surface of the scaffold respectively. (d) Pore area measurement using image. J (p = 0.0001). (e) In vitro cumulative drug release of dipyridamole from nanofibers containing various concentrations of drug (PCL/PDO + 5%DY, PCL/PDO + 7%DY, and PCL/PDO + 10%DY). The insert shows a higher magnification view of the area indicated with a red dotted circle. [file 13036_2022_313_MOESM5_ESM.docx]

Supplementary information

Rapid remodeling at mid-term *in vivo* study of a smart reinforced acellular vascular graft implanted on a rat model.

*Francis O Obiweluozor^,^ .^1ɸ^,* *Mukhammad Kayumov^2ɸ^, Yujin Kwak ^2^, Hwa-Jin Cho^3^,* [*Chan-Hee Park*](https://pubmed.ncbi.nlm.nih.gov/?term=Park+CH&cauthor_id=35167044) *^4^, Jun- kyu Park ^5^, Yun-Jin Jeong ^6^,Dong-Weon Lee ^6^, Do-Wan Kim*^2^, In-Seok Jeong*^2^*

^1^ Research and Business Development foundation, Chonnam National University, 77 Yongbong-ro, Yongbong-dong, Buk-gu, Gwangju 61186, Republic of Korea.

^2^ Department of Thoracic and Cardiovascular Surgery, Chonnam National University Hospital and Medical School, 160 Baekseo-ro, Dong-gu, Gwangju, 61469, Republic of Korea.

^3^ Department of Pediatrics, Chonnam National University Children's Hospital and Medical School, Gwangju, 61469, Republic of Korea.

^4.^ Department of Mechanical Engineering Graduate School, Chonbuk National University, 567 Baekje-daero, Deokjin-gu, Jeonju, 54896, Republic of Korea.

^5.^ CGBio Co. Ltd., 244 Galmachi-ro, Jungwon-u, Seongnam, 13211, Republic of Korea.

^6^School of Mechanical Engineering Chonnam National University, Gwangju 61469, Repubic of Korea.

***^*^ Correspondence can be sent to*** In Seok Jeong & Do-Wan Kim *(*[*isjeong1201@gmail.com*](mailto:isjeong1201@gmail.com)*; maskjoa@naver.com)*

*Tel: +82 1048554130,*

*ɸ- These authors contributed equally.*


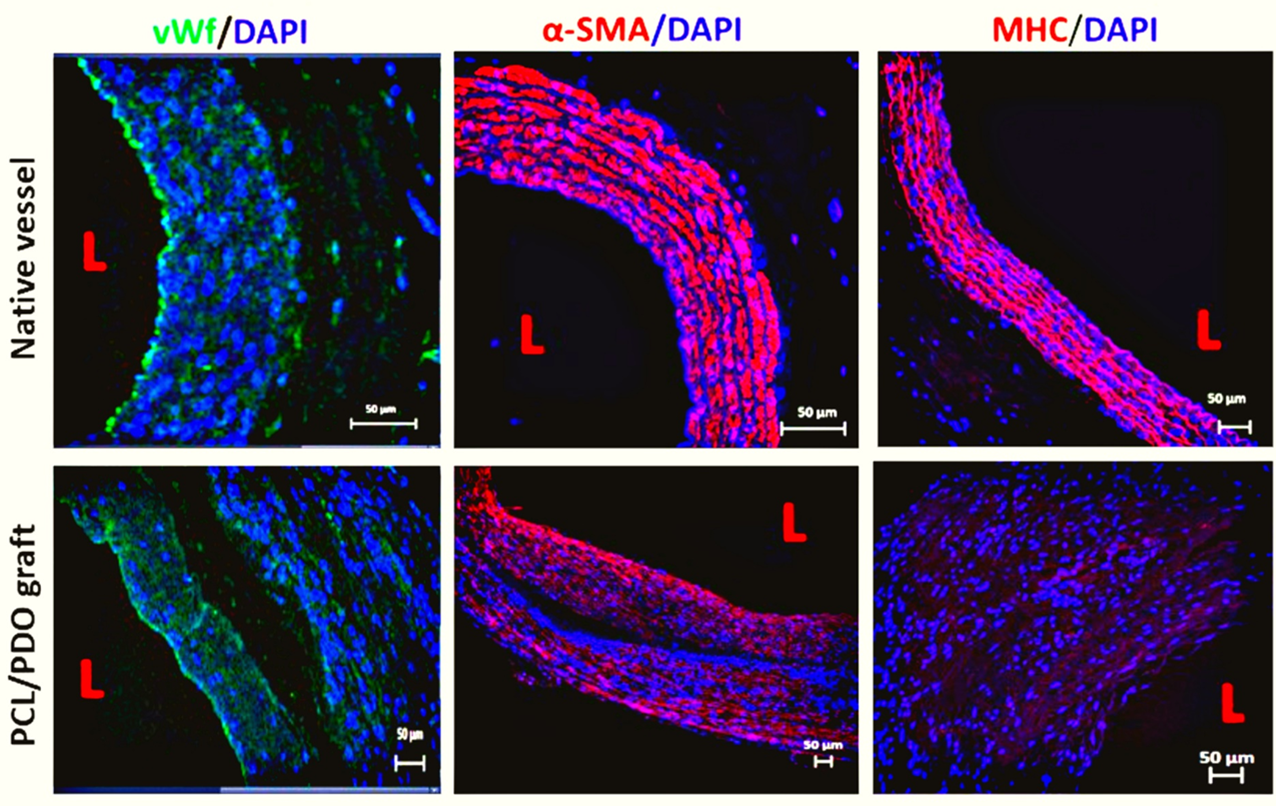


Figure 1: Tissue remodeling and extracellular matrix deposition in the explanted graft at 3 months as compared to native aorta. Cross-sectional images of native vessel and regenerated grafts (top row and bottom row respectively) were immunostained to examine the endothelial cells, smooth muscle cells, and myosin heavy chain. Endothelial cells were stained with vWF antibody (1^st^ column; green and Dapi stained blue). Smooth muscle cells were stained with α–smooth muscle antibody.


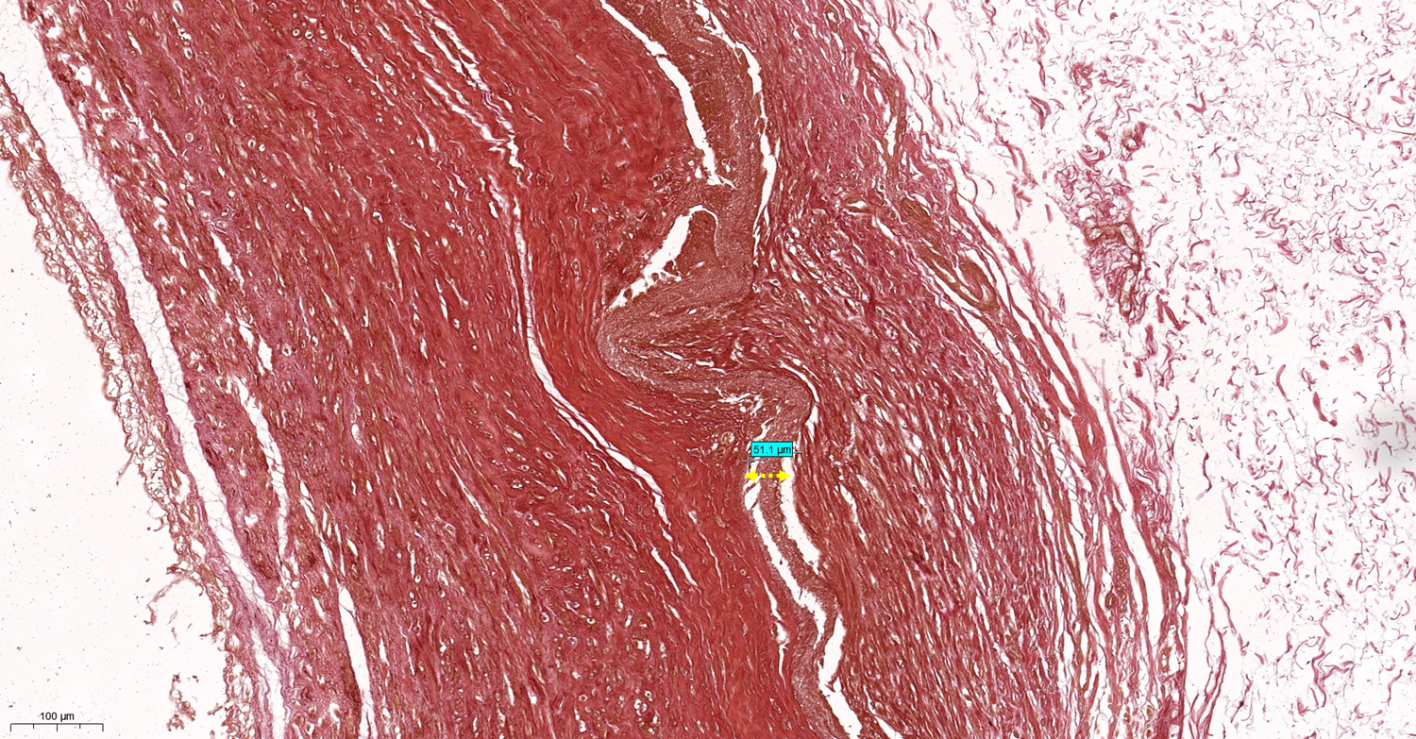


Figure 2: H&E staining of PCL/PDO graft at 12 weeks indicating remnants of PCL/PDO in the neo-vessel- yellow dotted arrow ( 51 µm). scale bar = 100 µm.


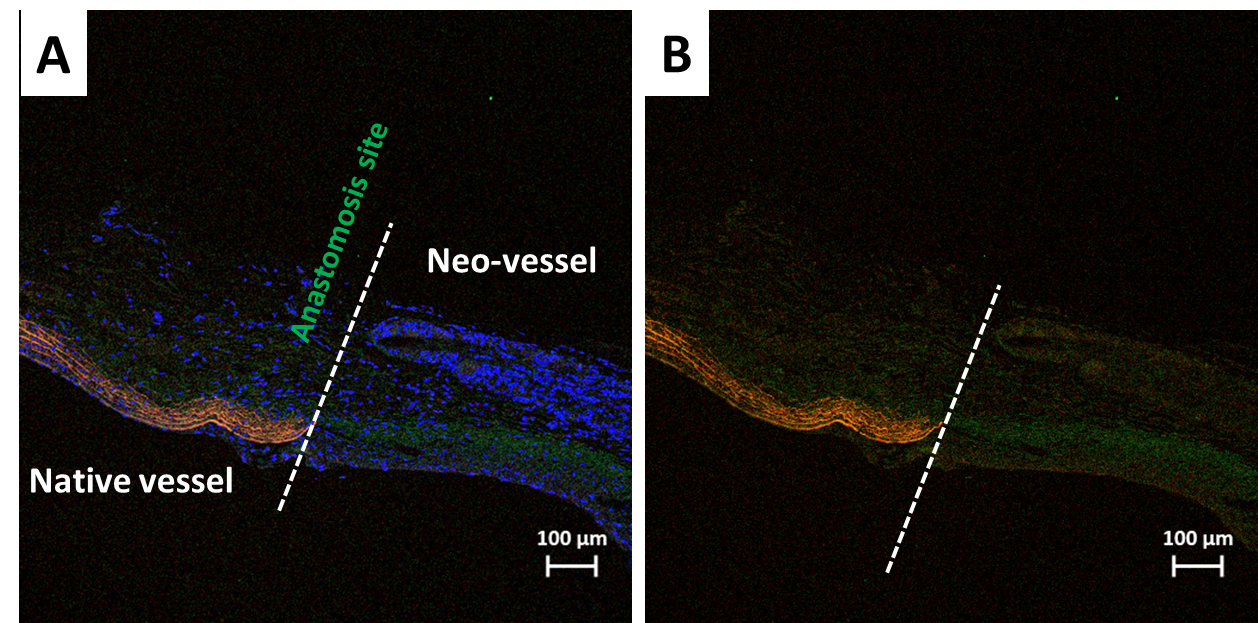


Figure 3: Longitudinal section of collagen stained PCL/PDO graft explant at 12 weeks stained. a) Collagen I & III staining was counterstained with Dapi-merge. b) Collagen I&III staining alone. White dotted line indicates anastomosis site.


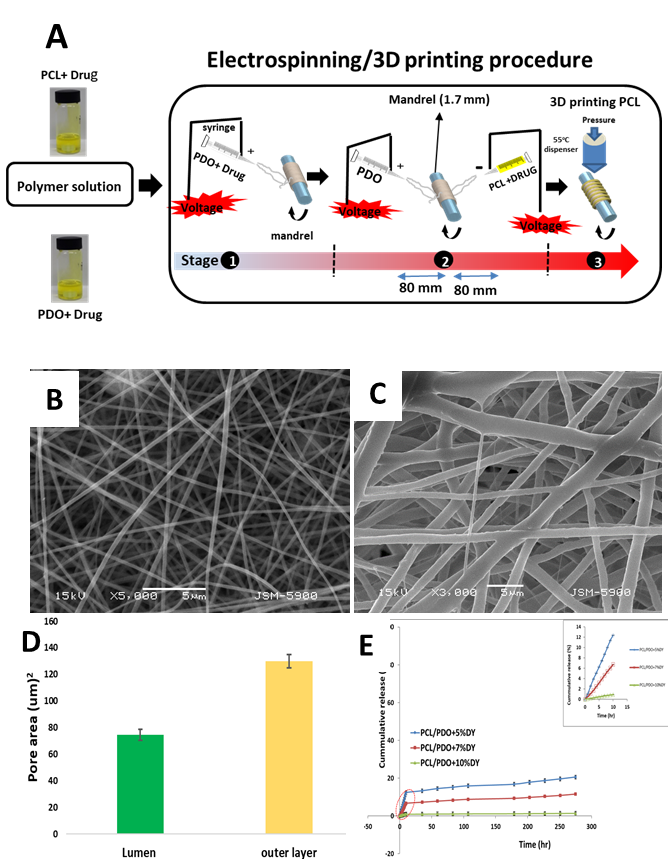


**Figure 4: (a) Fabrication process of graft. (b&c) Morphology of the luminal and outer surface of the scaffold respectively. (d) Pore area measurement using image. J (p= 0.0001). (e) In vitro cumulative drug release of dipyridamole from nanofibers containing various concentrations of drug (PCL/PDO+5%DY, PCL/PDO+7%DY, and PCL/PDO+10%DY). The insert shows a higher magnification view of the area indicated with a red dotted circle.**
